# Optimal cumulative cisplatin dose in nasopharyngeal carcinoma patients based on plasma Epstein–Barr virus DNA level after induction chemotherapy

**DOI:** 10.18632/aging.102920

**Published:** 2020-03-27

**Authors:** Sai-Lan Liu, Xue-Song Sun, Li-Ting Liu, Rui Sun, Dong-Hua Luo, Qiu-Yan Chen, Huan-Xin Lin, Li Yuan, Lin-Quan Tang, Ling Guo, Hai-Qiang Mai

**Affiliations:** 1 Sun Yat-Sen University Cancer Center, State Key Laboratory of Oncology in South China, Collaborative Innovation Center for Cancer Medicine, Guangzhou, Guangdong Province, People's Republic of China; 2 Department of Nasopharyngeal Carcinoma, Sun Yat-Sen University Cancer Center, Guangzhou, Guangdong Province, People's Republic of China; 3 Department of Radiotherapy, Sun Yat-Sen University Cancer Center, Guangzhou, Guangdong Province, People's Republic of China

**Keywords:** nasopharyngeal carcinoma, induction chemotherapy, EBV DNA, cumulative cisplatin dose

## Abstract

Purpose: This study aimed to elucidate the optimal cumulative cisplatin dose (CCD) for concurrent chemoradiotherapy (CCRT) according to the post-induction chemotherapy (IC) plasma Epstein–Barr virus (EBV) DNA level.

Results: EBV DNA was detected and undetected in 179 and 370 patients, respectively. Of the entire cohort, 73/549 (13.3%) patients received a total CCD ≥ 160 mg/m^2^ and 476/549 (86.7%) patients, <160 mg/m^2^. CCD enhancement was not associated with a survival benefit in patients with undetected EBV DNA after IC. However, among patients with post-IC detectable EBV DNA, higher 3-year PFS and locoregional relapse-free survival (LRFS) rates were observed in those who received a CCD ≥ 160 mg/m^2^. Multivariate analysis also showed CCD was an independent prognostic factor for PFS and LRFS in patients with post-IC detectable EBV DNA.

Conclusions: CCD enhancement was not associated with a survival benefit in patients with undetected EBV DNA after IC. However, among patients with post-IC detectable EBV DNA, those receiving ≥160 mg/m^2^ CCD showed significantly improved 3-year PFS and LRFS.

Methods: NPC patients (549) treated with IC and CCRT were included. Prognosis was assessed using a multivariate Cox proportional hazards model. Furthermore, grade 1–4 toxicities were compared between different CCD groups.

## INTRODUCTION

Nasopharyngeal carcinoma (NPC) is an endemic malignancy prevailing in Southern China and Southeast China [1, 2]. It is very strongly associated with Epstein–Barr virus (EBV) infection, with plasma EBV DNA being detectable in approximately 90% of cases [3]. Radiotherapy with concurrent cisplatin-based chemotherapy is the standard treatment regime for locoregionally advanced NPC [4–6]. Recently, several randomized controlled trials demonstrated induction chemotherapy (IC) prior to concurrent chemotherapy (CCRT) could afford a survival benefit for NPC patients in the era of intensity-modulated radiation therapy (IMRT) [7–9]. However, 20-30% of patients still show locoregional or distant relapse [10]. Hence, identifying patients with different risks of treatment failure and providing evidence for the need of individualized therapy are urgent.

Previous studies have reported that plasma EBV DNA levels after IC are closely correlated with the survival outcome of NPC patients and could be used to stratify patients in different risk groups [11, 12]. Patients with detectable EBV DNA level after IC are at a high risk of treatment failure and need intensive treatment. In contrast, in patients with undetectable EBV DNA level after IC, treatment intensity could be reduced to avoid unnecessary toxicities.

In terms of concurrent chemotherapy dosage, cisplatin-based regimens can be delivered at 30–40 mg/m^2^ per week or as one administration of 80-100 mg/m^2^ every 3 weeks [13, 14]. Optimal concurrent chemotherapy dosage administration is important in daily clinical practice. In this study, we aimed to investigate the prognostic value of cumulative cisplatin dose (CCD) in patients who underwent IC plus CCRT using IMRT. Furthermore, a subgroup analysis was conducted to compare the therapeutic value of CCD in patients in different risk groups. Our findings will help guide treatment strategies and modification of the CCRT regimen.

## RESULTS

From January 2009 to December 2017, a total of 549 NPC patients who underwent IC plus CCRT using IMRT were involved in the study. The median patient age was 43 years (range 8-77); 419 patients were male and 130 were female. Among them, 338 patients (61.6%) received TPF IC regimen, 145 patients (26.4%) received PF, and 66 patients (12.0%) received TP. After IC, EBV levels were undetectable and detectable in 370 patients (67.4%) and 179 patients (32.6%), respectively. Table 1 lists the characteristics of the 549 patients grouped by EBV DNA level after IC. Patients with advanced N stage, clinical stage, and higher pre-EBV DNA level were significantly inclined to have detectable EBV DNA levels after IC. There were no significant differences in other clinical characteristics between the two groups.

**Table 1:** Patient demographics and clinical characteristics.

**Characteristic**	**No. (%)**	**Undetectable EBV DNA (n = 370)**	**Detectable EBV DNA (n = 179)**	***P value***
		**No. (%)**	**No. (%)**	
**Age, years**				0.572^#^
Median (range)	43(8-77)	43(13-77)	44(8-74)	
< 45	288(52.5)	191(51.6)	97(54.2)	
≥45	261(47.5)	179(48.4)	82(45.8)	
**Sex**				0.934^#^
Female	130(23.7)	88(23.8)	42(23.5)	
Male	419(76.3)	282(76.2)	137(76.5)	
**Pathological type**				0.329^$^
WHO type I	2(0.4)	2(0.5)	0(0.0)	
WHO type II	3(0.5)	1(0.3)	2(1.1)	
WHO type III	544(99.1)	367(99.2)	177(98.9)	
T stage*				0.293^#^
T1	9(1.6)	6(1.6)	3(1.7)	
T2	65(11.8)	37(10.0)	28(15.6)	
T3	251(45.7)	172(46.5)	79(44.1)	
T4	224(40.8)	155(41.9)	69(38.5)	
N stage*				0.022^#^
N0	20(3.6)	15(4.1)	5(2.8)	
N1	134(25.6)	95(25.7)	39(21.8)	
N2	267(48.6)	188(50.8)	79(44.1)	
N3	128(23.3)	72(19.5)	56(31.3)	
Clinical stage*				0.024^#^
II	7(2.1)	5(1.4)	2(1.1)	
III	227(41.3)	161(43.5)	66(36.9)	
IVa	187(34.1)	132(35.7)	55(30.7)	
IVb	128(23.3)	72 (19.5)	56(31.3)	
**EBV DNA**				0.029^#^
<4000	273(49.7)	196 (53.0)	77(43.0)	
≥4000	276(50.3)	174 (47.0)	102(57.0)	
**IC regimen**				0.767^#^
TPF	338(61.6)	226(61.1)	112(62.6)	
PF	145(26.4)	101(27.3)	44(24.6)	
TP	66(12.0)	43(11.6)	23 (12.8)	
**Cisplatin regimen**			0.898^#^
3 weekly	477(86.9)	321(86.8)	156(87.2)	
Weekly	72(13.1)	49(13.2)	23(12.8)	
**CCD (mg/m^2^)**				0.163^#^
Median (range)	160(40-300)	160(40-300)	160(40-300)	
<160	73(13.3)	44(11.9)	29(16.2)	
≥160	476(86.7)	326(88.1)	150(83.8)	

Abbreviations: IC = induction chemotherapy; TPF = taxanes plus cisplatin with fluorouracil; PF = cisplatin with fluorouracil; TP = taxanes with cisplatin; EBV, Epstein–Barr virus; CCD = cumulative cisplatin dose during radiotherapy.

^#^P values were calculated by the Chi-square test. ^$^P value was calculated with Fisher’s exact test.

^*^According to the 7^th^ edition of the UICC/AJCC staging system.

### Relationship between EBV DNA level after IC and clinical outcome

**Figure 1:**
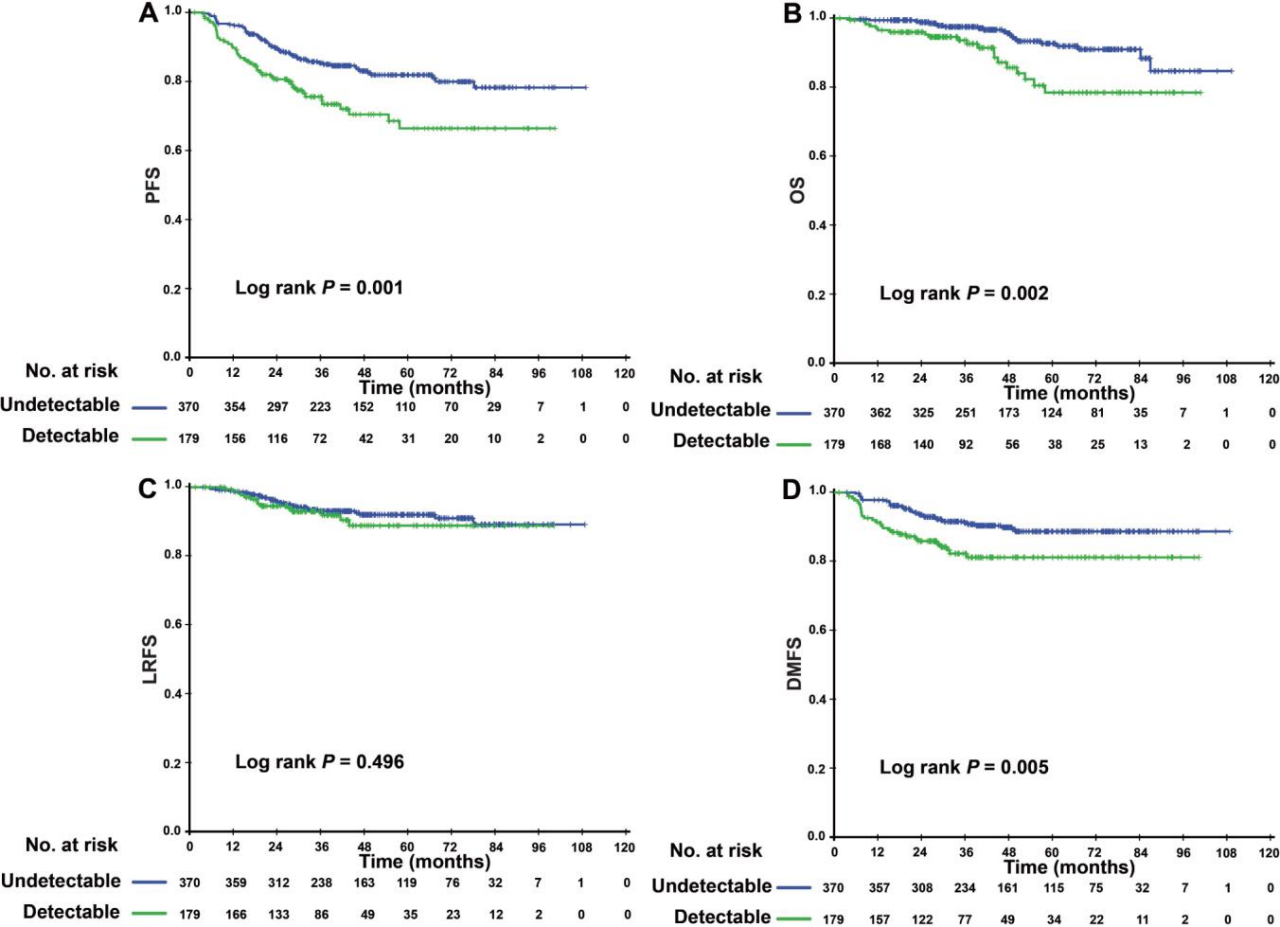
Kaplan–Meier PFS (**A**), LRFS (**B**), OS (**C**) and DMFS (**D**) curves for 549 patients with NPC with undetectable/detectable EBV DNA level after induction chemotherapy. Abbreviations: PFS = progression-free survival; OS = overall survival; LRFS = local-regional relapse-free survival; DMFS = distant metastasis-free survival; NPC, nasopharyngeal carcinoma; and EBV= Epstein–Barr virus.

The 3-year PFS of patients with a detectable EBV DNA level after IC was significantly worse than that of patients with undetectable EBV DNA level (74.5, 95% confidence interval [CI]: 67.4–81.6% versus 85.0, 95% CI: 81.1–88.9%, P = 0.001) (Figure 1A). The same trend was found in terms of OS and DMFS (3-year OS: 92.6% vs. 97.1%, P = 0.002; 3-year DMFS: 81.1% vs. 91.2%, P = 0.005) (Figure 1B, 1C). The 3-year LRFS did not significantly differ between these two groups: 91.8% (95% CI 87.3%- 96.3%) versus 93.0% (95% CI 90.3%-95.7%, P = 0.496) (Figure 1D). In the multivariate analysis, the levels of EBV DNA after IC were significantly associated with PFS (HR: 1.738, 95% CI: 1.173-2.576, P = 0.006), OS (HR: 1.482, 95% CI: 0.762-2.881, P = 0.008), and DMFS (HR: 1.891, 95% CI: 1.147-3.118, P = 0.013) (Table 2).

**Table 2:** Multivariate analysis of prognostic factors for 549 NPC patients receiving induction chemotherapy.

	Hazard ratio* (95% CI)	***P* value**
**Progression-free survival**		
Age (y) (≥ 45 vs. < 45)	0.739(0.496-1.102)	0.138
Gender (F vs. M)	1.111(0.704-1.753)	0.652
T category (3-4 vs. 1-2)	1.251(0.694-2.288)	0.456
N category (2-3 vs. 0-1)	0.814(0.529-1.253)	0.350
Overall stage (IVa-b vs. II-III)	1.001(0.671-1.496)	0.994
EBV DNA (≥4000 vs. <4000)	2.053(1.341-3.144)	0.001
EBV DNA (>0 vs. 0)	1.738(1.173-2.576)	0.006
CCD (≥160 vs. < 160)	0.699(0.419-1.164)	0.169
**Overall survival**		
Age (y) (≥ 45 vs. < 45)	1.466(0.781-2.752)	0.233
Gender (F vs. M)	0.997(0.484-2.054)	0.994
T category (3-4 vs. 1-2)	1.046(0.389-2.810)	0.929
N category (2-3 vs. 0-1)	0.643(0.335-1.234)	0.184
Overall stage (IVa-b vs. II-III)	1.657(0.843-3.256)	0.143
EBV DNA (≥4000 vs. <4000)	1.482(0.762-2.881)	0.246
EBV DNA (>0 vs. 0)	2.345(1.252-4.393)	0.008
CCD (≥160 vs. < 160)	0.962(0.401-2.308)	0.930
**Locoregional relapse-free survival**		
Age (y) (≥ 45 vs. < 45)	0.706(0.370-1.346)	0.290
Gender (F vs. M)	1.191(0.577-2.459)	0.637
T category (3-4 vs. 1-2)	2.071(0.615-6.972)	0.240
N category (2-3 vs. 0-1)	0.522(0.272-1.002)	0.051
Overall stage (IVa-b vs. II-III)	0.931(0.493-1.758)	0.826
EBV DNA (≥4000 vs. <4000)	2.843(1.388-5.823)	0.004
EBV DNA (>0 vs. 0)	1.097(0.568-2.118)	0.782
CCD (≥160 vs. < 160)	0.456(0.222-0.939)	0.033
**Distant metastasis-free survival**		
Age (y) (≥ 45 vs. < 45)	0.736(0.440-1.230)	0.243
Gender (F vs. M)	1.070(0.594-1.927)	0.821
T category (3-4 vs. 1-2)	1.116(0.538-2.313)	0.768
N category (2-3 vs. 0-1)	1.106(0.615-1.989)	0.736
Overall stage (IVa-b vs. II-III)	0.904(0.543-1.505)	0.698
EBV DNA (≥4000 vs. <4000)	1.801(1.051-3.085)	0.032
EBV DNA (>0 vs. 0)	1.891(1.147-3.118)	0.013
CCD (≥160 vs. < 160)	1.044(0.496-2.196)	0.911

Abbreviations: CI = confidence interval; EBV= Epstein–Barr virus; IC = induction chemotherapy.

### Relationship between CCD and clinical outcome

When a different CCD was considered as a prognostic factor, we found that patients who received a higher total CCD (≥160) achieved a higher 3-year LRFS (93.4% vs. 87.3%) in comparison with those who received a lower total CCD. However, there were no significant differences in the other three clinical endpoints (Supplementary Figure 1). Multivariate analysis also demonstrated that a higher CCD predicted a better 3-year LRFS rate than a lower CCD (HR 0.456, 95% CI 0.222-0.939, P = 0.033).

### The efficacy of CCD in patients with different EBV DNA levels after IC

**Figure 2:**
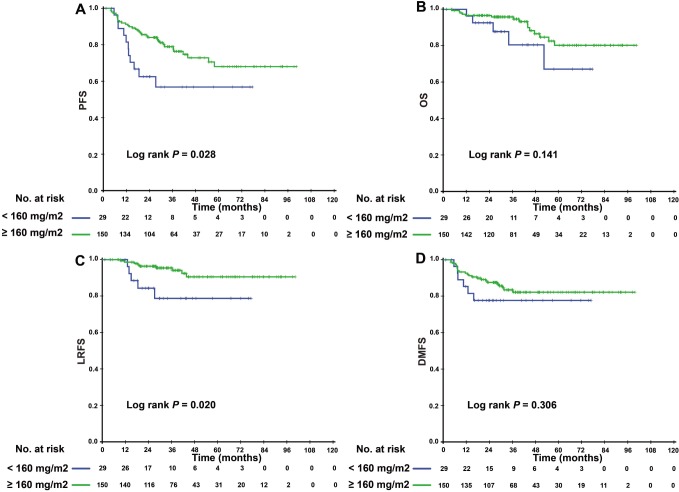
Kaplan–Meier PFS (**A**), OS (**B**), LRFS (**C**), and DMFS (**D**) curves for the subgroup of 174 NPC patients with detectable EBV DNA level after induction chemotherapy stratified by CCD < 160 mg/m^2^, and CCD ≥ 160 mg/m^2^. Abbreviations: PFS = progression-free survival; OS = overall survival; LRFS = local-regional relapse-free survival; DMFS = distant metastasis-free survival; NPC, nasopharyngeal carcinoma; CCD = cumulative cisplatin dose.

**Figure 3:**
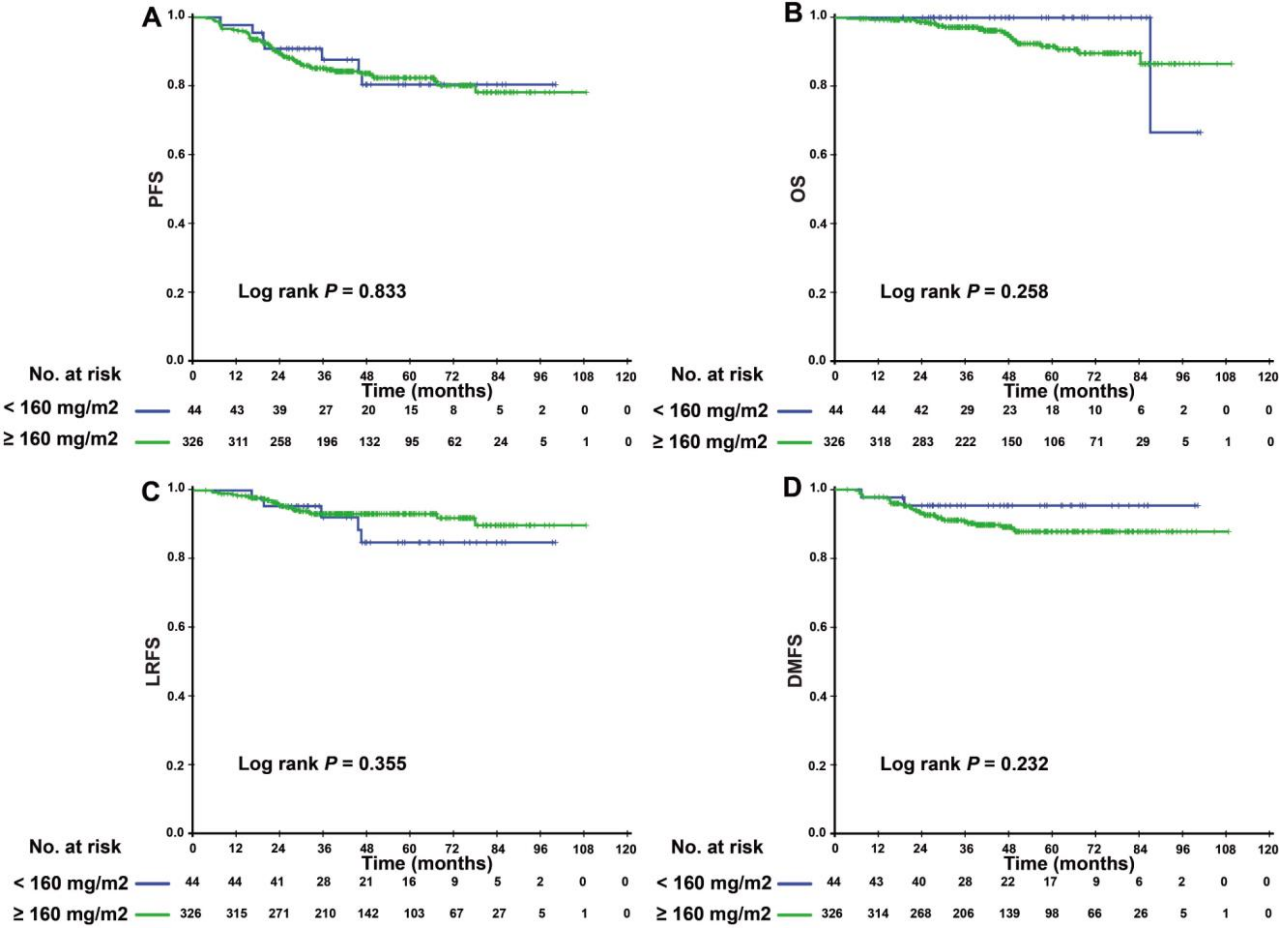
Kaplan–Meier PFS (**A**), OS (**B**), LRFS (**C**), and DMFS (**D**) curves for the subgroup of 370 NPC patients with undetectable EBV DNA level after induction chemotherapy stratified by CCD < 160 mg/m^2^, and CCD ≥ 160 mg/m^2^. Abbreviations: PFS = progression-free survival; OS = overall survival; LRFS = local-regional relapse-free survival; DMFS = distant metastasis-free survival; NPC, nasopharyngeal carcinoma; CCD = cumulative cisplatin dose.

Since patients with detectable or undetectable EBV DNA after IC showed different tumor burdens and clinical prognoses, we further explored the efficacy of CCD in patients with different EBV DNA levels after IC. Interestingly, we found that CCD played different roles in relation to its efficacy in these two subgroups. In patients with detectable EBV DNA, higher CCD was significantly correlated with a higher 3-year OS rate and LRFS (3-year PFS: 76.4% vs. 56.8%, P = 0.028; 3-year LRFS: 94.1% vs. 78.6%, P = 0.020) (Figure 2). In multivariate analysis, Table 3 showed that CCD remained an independent prognostic factor for PFS (HR 0.497, 95% CI 0.250–0.988, P = 0.046) and LRFS (HR 0.325, 95% CI 0.106–0.993, P = 0.049). However, a higher CCD did not yield significant survival benefits for patients with undetectable EBV DNA levels after IC. The Kaplan–Meier survival curves are shown in Figure 3.

**Table 3:** Multivariate analysis of prognostic factors for 179 NPC patients with detectable EBV DNA after induction chemotherapy.

	Hazard ratio* (95% CI)	***P* value**
**Progression-free survival**		
Age (y) (≥ 45 vs. < 45)	1.028(0.561-1.883)	0.930
Gender (F vs. M)	1.053(0.516-2.151)	0.887
T category (3-4 vs. 1-2)	0.985(0.444-2.186)	0.971
N category (2-3 vs. 0-1)	0.847(0.433-1.657)	0.628
Overall stage (IVa-b vs. II-III)	1.044(0.557-1.956)	0.894
EBV DNA (≥4000 vs. <4000)	2.160(1.068-4.368)	0.032
CCD (≥160 vs. < 160)	0.497(0.250-0.988)	0.046
**Overall survival**		
Age (y) (≥ 45 vs. < 45)	1.295(0.512-3.275)	0.586
Gender (F vs. M)	0.827(0.294-2.322)	0.718
T category (3-4 vs. 1-2)	0.650(0.199-2.120)	0.475
N category (2-3 vs. 0-1)	0.601(0.234-1.541)	0.289
Overall stage (IVa-b vs. II-III)	1.738(0.637-4.737)	0.280
EBV DNA (≥4000 vs. <4000)	1.127(0.430-2.955)	0.807
CCD (≥160 vs. < 160)	0.508(0.180-1.433)	0.201
**Locoregional relapse-free survival**		
Age (y) (≥ 45 vs. < 45)	1.478(0.506-4.313)	0.475
Gender (F vs. M)	0.712(0.219-2.312)	0.572
T category (3-4 vs. 1-2)	1.028(0.205-5.144)	0.973
N category (2-3 vs. 0-1)	0.538(0.169-1.713)	0.294
Overall stage (IVa-b vs. II-III)	2.167(0.571-8.221)	0.256
EBV DNA (≥4000 vs. <4000)	9.197(1.163-72.750)	0.035
CCD (≥160 vs. < 160)	0.325(0.106-0.993)	0.049
**Distant metastasis-free survival**		
Age (y) (≥ 45 vs. < 45)	1.045(0.494-2.211)	0.909
Gender (F vs. M)	1.107(0.442-2.773)	0.828
T category (3-4 vs. 1-2)	1.093 (0.404-2.952)	0.861
N category (2-3 vs. 0-1)	1.063(0.440-2.567)	0.892
Overall stage (IVa-b vs. II-III)	0.782(0.367-1.665)	0.523
EBV DNA (≥4000 vs. <4000)	1.903(0.819-4.421)	0.135
CCD (≥160 vs. < 160)	0.660(0.266-1.636)	0.369

Abbreviations: CI = confidence interval; EBV= Epstein–Barr virus; IC = induction chemotherapy.

### Acute toxicity

We then evaluated the acute toxicity associated with different CCDs. The number of patients with grade 1, 2, 3, and 4 toxicities is shown in Supplementary Table 1. Intergroup differences in hematological toxicities such as leukocytopenia (83.6% vs. 86.6%), neutropenia (82.2% vs. 69.4%), anemia (78.1% vs. 83.2%), and thrombocytopenia (24.7% vs. 23.9%) were not significant (P > 0.05 for all comparisons; Supplementary Table 1). In addition, patients with different CCDs showed similar levels of hepatotoxicity [ALT increase (16.5% vs. 26.6%) and AST increase (12.3% vs. 9.0%)]. However, a higher incidence of grade 1-4 nephrotoxicity was observed in the group with CCD ≥ 160 (28.8% vs. 43.0%; P = 0.021).

## DISCUSSION

Our study revealed that detectable EBV DNA levels after IC were associated with significantly improved PFS, OS, and DMFS in NPC patients. In the subgroup of patients with detectable EBV DNA levels after IC, patients receiving a total CCD < 160 mg/m^2^ showed significantly decreased 3-year PFS and LRFS in A Cox proportional hazards regression model was used to detect variables individually without adjustment. All variables were transformed into categorical variables. HRs were calculated for age (years: ≥45 vs. <45), sex (female vs. male), T stage (T3-4 vs. T1-2), N stage (N2-3 vs. N0-1), plasma EBV DNA before the first treatment (≥4000 copies/ml vs. <4000 copies/ml), plasma EBV DNA after IC (>0 copies/ml vs. 0 copies/ml), and overall stage (IVa-b vs. II-III).

comparison with patients receiving a total CCD ≥ 160mg/m^2^. However, in the subgroup of patients with undetectable EBV DNA levels after IC, there were no significant differences in survival endpoints between patients receiving different CCD regimens.

Several studies have demonstrated that IC confers a survival benefit in locally advanced NPC patients [7–9]. However, a small proportion of patients did not respond to cisplatin-based IC. In previous studies, the percentages of patients with detectable EBV DNA levels after IC ranged from 23.7%-33.8% [11, 15, 16]. In our study, EBV DNA levels after IC were detectable in 32.6% of patients (179 of 549), which was consistent with previous results. In addition, previous studies proved that the EBV DNA load after IC is an earlier and powerful prognostic factor in patients with NPC [11, 16]. Our results are in good agreement with the findings of these studies, which demonstrated that the EBV DNA load after IC was an independent prognostic factor for PFS, OS, and DMFS, but not LRFS. Specifically, patients with detectable EBV DNA after IC most frequently experienced distant failure. The EBV DNA load after IC may be useful for risk stratification and early identification of higher-risk patients before CCRT to guide clinicians in adjusting the intensity of concurrent chemoradiotherapy as early as possible to improve the therapeutic effect and eventually improve survival.

Cisplatin-based concurrent chemotherapy administered during RT is important in conferring survival benefits [17, 18]. Many previous studies have reported the prognostic value of CCD, and a CCD of 200 mg/m^2^ has been shown to significantly improve the prognosis in NPC patients receiving CCRT [14, 19–22]. In addition, Peng et al. retrospectively assessed the relationship between the CCD and the prognosis of NPC patients receiving CCRT without IC, which found that CCD (≥240 mg/m2) had no prognostic value in subgroup analysis with stratification by the cut-off value of pre-DNA (1460 copies/ml). However, all patients in our study received IC+CCRT and we did subgroup analysis based on the EBV DNA load after IC, which distinguished from their study [23]. Lv and colleagues found that a CCD of 200 mg/m^2^ did not yield significant improvements in survival outcomes in NPC patients receiving IC plus CCRT, while a CCD of 160 mg/m^2^ A Cox proportional hazards regression model was used to detect variables individually without adjustment. All variables were transformed into categorical variables. HRs were calculated for age (years: ≥45 vs. <45), sex (female vs. male), T stage (T3-4 vs. T1-2), N stage (N2-3 vs. N0-1), plasma EBV DNA before the first treatment (≥4000 copies/ml vs. <4000 copies/ml), and overall stage (IVa vs. II-III).

might be enough to yield beneficial antitumor effects [24]. However, their study sample was relatively small, and the prognostic difference was discussed only for the whole cohort and subgroup according to the EBV DNA load after IC has never been done before, which could distinguish patients with locoregionally advanced NPC into a low-risk group (with undetectable EBV DNA) and a high-risk group (with detectable EBV DNA). Patients with a detectable EBV DNA load after IC who have a higher tumor burden had a higher risk of recurrence and metastasis in the future, which indicates that they should receive intensification of therapy, and our results demonstrated that an increasing CCD seemed to achieve better local control for those higher-risk patients. Although there was an association between CCD and PFS and LRFS in subgroups of patients with detectable EBV DNA load after IC, the prognostic effect was not observed in terms of OS and DMFS. Our results were consistent with the study conducted by Lee et al., which confirmed that concurrent chemoradiotherapy could significantly improve tumor control, particularly at locoregional sites. In addition, our research proved that there were no significant differences in survival endpoints between patients receiving the different regimens of CCD in the low-risk group. For the low-risk group with a better prognosis, reducing the treatment intensity could be taken into consideration to avoid unnecessary toxicities and cost. The initial treatment of NPC is very important, and determining the appropriate course of chemotherapy is crucial, while the EBV DNA load after completion of IC is an ideal marker providing an adequate basis for determining the optimal treatment regime. Therefore, it is possible that the EBV DNA load after IC could guide clinicians in adjusting concurrent chemotherapy treatment intensity to improve the therapeutic effect and finally improve survival as early as possible before CCRT.

These findings provide us with a new clinical implication that the EBV DNA load after IC should be considered as an important stratification factor for the design of different intensive concurrent chemotherapies in patients with lower or higher viral loads in future trials. Selecting the treatment strategy for IC based on the EBV DNA level may enable further improvement in NPC prognosis. In our opinion, routine delivery of three courses of cisplatin-based concurrent chemotherapy (80-100 mg/m^2^) or six courses (30-40 mg/m^2^) for “all” advanced-stage NPC patients should be reconsidered. Indeed, patients with advanced disease included a heterogeneous group with variable relapse rate. Pretreatment prognostic factors (patient characteristics and initial clinical stages) are no longer important because the tumors in most patients are mostly eradicated after initial IC. As IC continues, the EBV DNA load after IC may reflect the tumor response to treatment and dynamically select the subgroup with a high risk of distant metastasis; thus, it is a potential predictor to guide clinicians to modify treatment strategy in a timely manner. On the other hand, immunotherapy and targeted therapy could also be taken into consideration for patients with EBV DNA loads after IC to improve the therapeutic effect and finally improve survival on the basis of increasing the concurrent cisplatin dose.

Nevertheless, the present study has several limitations. First, there was an inevitable selection bias caused by the retrospective nature of the study. Second, the data were obtained exclusively from one center; therefore, these results must be validated by other institutions. Third, the follow-up duration of our study was short. It is necessary to use longer follow-up periods to both evaluate the long-term outcomes of these patients and validate our results.

## CONCLUSIONS

Plasma EBV DNA level after IC is an independent prognostic factor for patients with NPC. Enhancement of CCD was not associated with a survival benefit in patients with undetected EBV DNA after IC. However, among patients who showed EBV DNA after IC, those receiving ≥160 mg/m^2^ CCD showed significantly improved 3-year PFS and LRFS than those receiving <160 mg/m^2^ CCD. Our data suggest that increasing the CCD could improve the efficacy for these patients.

## MATERIALS AND METHODS

### Patients

From 2009 to 2017, 549 patients with local advanced NPC at Sun Yat-sen University Cancer Center, China, were included in our study. The eligibility criteria for the study were as follows: (1) pathologically confirmed NPC of stages II-IV; (2) Karnofsky performance score (KPS) of >70; (3) treated using IMRT; (4) treatment with first-line IC and cisplatin-based concurrent chemotherapy regimen; (5) availability of EBV DNA data after IC; and (6) adequate organ function. The exclusion criteria were as follows: any history of malignancy; received previous anti-tumor treatment; received palliative treatment; the presence of pregnancy, lactation or severe coexisting illness.

### Pretreatment evaluation

All patients underwent a complete physical examination, fiberoptic nasopharyngoscopy, magnetic resonance imaging (MRI) of the nasopharynx and neck, chest radiography, bone scan or whole-body fluorodeoxyglucose positron emission tomography (PET)/computed tomography (CT), complete blood count, renal and liver function tests, and plasma EBV DNA level determination.

### Plasma EBV DNA level assessment

Plasma EBV DNA concentrations were measured by real-time quantitative polymerase chain reaction before treatment and after IC [25, 26]. The cutoff values for pretreatment EBV DNA level and EBV DNA level after IC were 4000 copies/mL and 0 copy/mL, respectively, which has been established as a prognostic value in previous studies [11, 27].

### Treatment and evaluation

All patients received one of the following IC regimens: PF (consisting of cisplatin [1 day of 80-100 mg/m^2^] and 5-fluorouracil [800-1000 mg/m^2^, by 120 h of continuous intravenous infusion]), TP (consisting of docetaxel [1 day of 75 mg/m^2^] or paclitaxel [1 day of 150-180 mg/m^2^] or paclitaxel liposome [1 day of 150-180 mg/m^2^] and cisplatin [20-25 mg/m^2^ on days 1-3]) and TPF (consisting of docetaxel [1 day of 60 mg/m^2^] or paclitaxel [1 day of 135 mg/m^2^] or paclitaxel liposome [1 day of 135 mg/m^2^], cisplatin [1 day of 60 mg/m^2^], and 5-fluorouracil [500-800 mg/m^2^, by 120 h of continuous intravenous infusion]). All regimens were administered at intervals of 3 weeks for 2-4 cycles.

All patients received IMRT. Gross tumor volume included the primary tumor and the positive retropharyngeal lymph node. A total dose of 68-70 Gy was administered with the daily fraction ranging from 2.00 Gy to 2.34 Gy. Other details of the IMRT plan were in accordance with previous studies [28–30]. Concurrent cisplatin-based chemotherapy (80-100 mg/m^2^ every 3 weeks or 30-40 mg/m^2^ weekly) was administered during radiotherapy [18, 31].

### Outcome and follow-up

Our primary study endpoint was progression-free survival (PFS), which was calculated from the first day of treatment to the date of any treatment failure or death from any cause. The secondary endpoints included overall survival (OS), which was calculated from the first day of treatment to the date of death from any cause; distant metastasis-free survival (DMFS), which was calculated from the first day of treatment to the date of distant metastasis; and locoregional relapse-free survival (LRFS), which was calculated from the first day of treatment to the date of locoregional failure. After treatment, the patients were examined every 3 months for the first 3 years and every 6 months thereafter or until death.

### Statistical analysis

All statistical analyses in our study were performed using SPSS package for Windows, version 22.0 (Chicago, IL). Categorical variables were compared by Chi-square test or Fisher’s exact test. Survival parameters were calculated using Kaplan–Meier actuarial analysis and differences were compared using the log-rank test. Multivariate analyses were performed using the Cox proportional hazards regression model. All analyses were two-sided. The level of significance was set at P < 0.05.

### Ethics approval

This retrospective study was approved by the Clinical Research Committee of Sun Yat-sen University Cancer Center. Patients were required to provide written informed consent before enrolling in the study.

## Supplementary Material

Supplementary Figure 1

Supplementary Table 1
