# Supplementary material for: Optimal cumulative cisplatin dose in nasopharyngeal carcinoma patients based on plasma Epstein–Barr virus DNA level after induction chemotherapy
Source: Aging (Albany NY). 2020 Mar 27;12(6):4931–44. doi: 10.18632/aging.102920 (PMC7138583; doi:10.18632/aging.102920)
Supplement: Supplementary Figure 1 [file aging-12-102920-s001..pdf]

SUPPLEMENTARY FIGURE

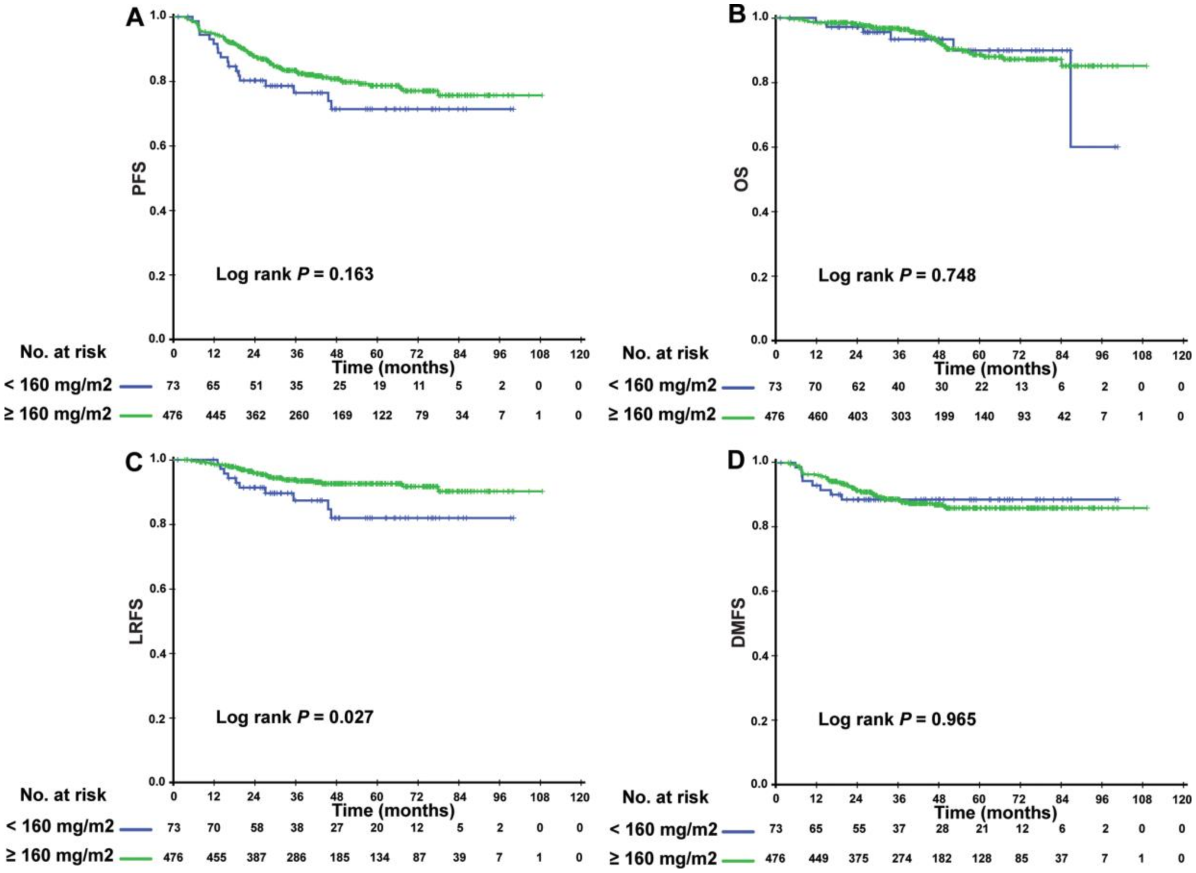

Supplementary Figure 1. Kaplan–Meier PFS (**A**), OS (**B**), LRFS (**C**), and DMFS (**D**) curves for 549 NPC patients stratified by CCD < 160 mg/m^2, and CCD ≥ 160 mg/m^2. Abbreviations: PFS = progression-free survival; OS = overall survival; LRFS = local-regional relapse-free survival; DMFS = distant metastasis-free survival; NPC, nasopharyngeal carcinoma; CCD = cumulative cisplatin dose.
