# Supplementary material for: Optimal cumulative cisplatin dose in nasopharyngeal carcinoma patients based on plasma Epstein–Barr virus DNA level after induction chemotherapy
Source: Aging (Albany NY). 2020 Mar 27;12(6):4931–44. doi: 10.18632/aging.102920 (PMC7138583; doi:10.18632/aging.102920)
Supplement: Supplementary Table 1 [file aging-12-102920-s002..pdf]

SUPPLEMENTARY TABLE

Supplementary Table 1. Grade 1–4 acute toxicities due to CCRT between the different CCD groups.

Adverse event	CCD < 160 (n=73)				CCD≥160 (n=476)				p value for events grade ≥1
(toxicity grade)	1(%)	2(%)	3 (%)	4(%)	1(%)	2(%)	3 (%)	4(%)	
Leucocytopenia	17(23.3)	30(41.1)	14(19.2)	0(0.0)	99(20.8)	206(43.3)	100(21.0)	7(1.5)	0.491
Neutropenia	21(28.8)	19(26.0)	9(26.0)	1(1.4)	142(29.8)	134(28.2)	44(9.2)	15(2.2)	0.688
Anemia	35(47.9)	21(28.8)	0(0.0)	1(1.4)	213(44.7)	145(30.5)	30(6.3)	8(1.7)	0.284
Thrombocytopenia	10(13.7)	3(4.1)	4(5.5)	1(1.4)	61(12.8)	38(8.0)	12(2.5)	3(0.6)	0.895
AST increase	5(6.8)	3(4.1)	1(1.4)	0(0.0)	37(7.8)	4(0.8)	1(0.2)	1(0.2)	0.371
ALT increase	8(11.0)	1(1.4)	3(4.1)	0(0.0)	114(23.9)	10(2.1)	2(0.4)	1(0.2)	0.061
BUN increase	21(28.8)	0(0.0)	0(0.0)	0(0.0)	201(42.2)	3(0.6)	0(0.0)	1(0.2)	0.021
Creatinine increase	11(15.1)	1(1.4)	0(0.0)	0(0.0)	114(23.9)	2(0.4)	1(0.1)	0(0.0)	0.127

Abbreviations: CCRT = concurrent chemoradiotherapy; CCD = cumulative cisplatin dose; ALT = alanine aminotransferase; AST = aspartate aminotransferase; BUN = blood urea nitrogen.
P values were calculated by Chi-square test.
